# The Evolution of Alexia in Two Cases of Posterior Cortical Atrophy

**DOI:** 10.3233/BEN-2011-0334

**Published:** 2011-08-29

**Authors:** Eleonora Catricalà, Pasquale A. Della Rosa, Paola Ortelli, Valeria Ginex, Alessandra Marcone, Daniela Perani, Stefano F. Cappa

**Affiliations:** ^1^Department of PsychologyMilano-Bicocca UniversityMilanItaly; ^2^Vita-Salute University and San Raffaele Scientific InstituteMilanItaly; ^3^Department of Clinical NeurosciencesSan Raffaele Turro HospitalMilanItaly; ^4^Nuclear Medicine Unit and Division of NeuroscienceSan Raffaele Scientific InstituteMilanItaly

**Keywords:** Alexia, Posterior Cortical Atrophy, ventral, dorsal

## Abstract

Posterior cortical atrophy (PCA) is an uncommon presentation of Alzheimer's disease (AD), characterised by prevalent anatomo-functional involvement of posterior cortical areas. Accordingly, the main clinical features at onset are disorders of high-order visual processing, such as alexia and impairments of visuo-spatial and visuo-constructional abilities. The clinical features in the early stages of disease are variable, and they have been suggested to stem from prevalent ventral or dorsal brain pathology, and/or asymmetric hemispheric involvement. With disease progression, these differences tend to blur with the increasing severity of neuropsychological dysfunction. We report two PCA patients showing different patterns of reading impairment (respectively, letter-by-letter reading and neglect dyslexia). A follow-up study suggested that the qualitative features of alexia remain distinctive with disease evolution. In addition, single photon emission tomography (SPECT) studies revealed different patterns of hypoperfusion, consistent with the alexia types. A careful reading assessment can provide important insights to the pattern of progression of the disease in patients with PCA up to the late stages of the pathology.

## 1. Introduction

Posterior Cortical Atrophy (PCA) is an uncommon neurodegenerative disorder, which is due to progressive dysfunction of the retrorolandic associative cortex. Clinically, PCA is characterised by impairments of high-order visual processing [8,20,30], and the course of the disease is variable in terms of progression of neuropsychological deficits. The possible symptoms include alexia, visual agnosia, agraphia, transcortical sensory aphasia, apraxia, visuo-spatial disorders, elements of Balint's syndrome (simultanagnosia, optical ataxia and optical apraxia) or of Gerstmann's syndrome (acalculia, right/left disorientation, finger agnosia, agraphia). In addition most patients develop visual field defects or visual hemineglect, which can fluctuate during the course of illness [7,12]. The underlying neuropathology appears to be due to Alzheimer's disease (AD) [2,29].

The clinical presentation of PCA is not homogeneous, and it has been proposed that different clinical pictures may reflect specific patterns of brain involvement. A distinction between a dorsal, parieto-occipital, and a ventral, temporo-occipital variant of PCA has been proposed on the basis of structural (Magnetic Resonance Imaging, MRI) and functional (single-photon emission computed tomography, SPECT) differences [25,26]. The dorsal variant is characterized by visuospatial and visuomotor dysfunction, leading to Balint's syndrome, Gerstmann's syndrome, dressing apraxia and transcortical sensory aphasia. The ventral variant is underpinned by alexia, visual agnosia and prosopagnosia. A third variant, with prominent involvement of primary visual cortex, has been characterized by Galton et al. [15]. In many patients there is evidence of both dorsal and ventral involvement [9,19, 30].

ISSN 0953-4180/11/$27.50 © 2011 – IOS Press and the authors. All rights reserved

An additional dimension of heterogeneity is hemispheric prevalence. Asymmetric structural and functional changes affecting one hemisphere more than the other have been reported by some investigators [5,17, 18,24,28]. A right hemispheric involvement may give rise to left visual hemi-neglect or hemi-agnosia [3,24]. In contrast, a prevalent left-sided involvement could instead lead to Gerstmann's syndrome or right hemi-achromatopsia and pure alexia [13,14]. In some cases the different variants may be identified at disease onset, but they tend to merge at later stages; namely the degeneration may progress from parietal to temporal areas [26], or may spread asymmetrically, involving both hemispheres [24].

PCA patients often complain of reading difficulties and the qualitative pattern of the reading impairment may be a potential neuropsychological marker of the pathological correlation. This symptom is probably the most common and one of the earliest in PCA [21]. However, the specific features of alexia and their evolution have been seldom investigated. A number of reports refer to the reading difficulties in PCA patients as "pure alexia" [1,9,13,14,16], but several varieties of alexia have been described in PCA patients, such as neglect alexia, alexia with simultanagnosia [20], "apperceptive" [22], attentional alexia [27], or a central reading disorder with lexical and spatial agraphia [3]. This study aims at highlighting the clinical evolution of different types of alexia in two PCA patients, one with a neuropsychological picture compatible with an initial and unilateral involvement (left) of the ventral areas, and the other with features of the dorsal variant and a prominent involvement of right hemisphere. They underwent examination, respectively, at two and three stages in the course of their illness and were submitted to MRI and regional cerebral blood flow (rCBF) SPECT studies.

## 2. Case reports

### 2.1. Case 1: L.P.

#### 2.1.1. First examination

A 66 year old right-handed man, with 13 years of education, self-employed in a construction firm, in 2005 showed reading problems (his family reported a syllable by syllable reading), driving difficulties (deviations toward the left) and mental calculation difficulties over the previous 7–8 months. The neurological examination indicated the presence of right hemianopia on confrontation testing. His Mini-Mental State Examination (MMSE [11]) score was 28/30. The neuropsychological assessment revealed acalculia and a reading disorder, with a letter by letter strategy, evident in particular for pseudowords and for long words. He scored 92/92 (100%) on a word reading task, 41/45 (91,1%) on a non word reading task (BADA [23]), where he made visual errors, for example "cafena" for "cofena", but no lexicalizations. Spontaneous writing and writing to dictation were preserved (45/46 (97,8%) for words and 25/25 (100%) for non-words). Language was fluent with good comprehension and repetition. Picture naming and controlled associations (phonemic and semantic) were within normal limits. His memory was normal. No problems in visuo-spatial and constructional abilities were detected. On tests assessing object perception abilities his score was above the cut-off point for normal performance (Visual Object and Space Perception Battery, VOSP [31]).

MRI revealed an enlargement of subarachnoid spaces in bilateral occipital and parietal regions and left posterior temporal cortex. The rCBF single-photon emission tomography (SPECT) voxel-based analysis showed a hypoperfusion limited to the left hemisphere and involving the inferior and middle temporal gyri and the middle occipital gyrus (Table 1).

In summary, L.P. showed prominent symptoms due to the involvement of the left hemisphere, in particular of the inferior occipito-temporal pathways. In addition, he showed a calculation disorder, which may reflect the involvement of the inferior parietal lobe (see MRI results).

#### 2.1.2. Second examination

3 years and 3 months later, L.P. reported that visual disturbances continued to be his major problem; his driving difficulties became more severe, and he was involved in 2 car accidents in 4 months. In addition he complained of memory and word-finding difficulties. His MMSE score was 24/30. The neuropsychological assessment revealed a marked worsening of the calculation disorder and alexia. However, on quantitative testing the reading performance remained almost the same as at the time of the first assessment, with a score of 91/92 (98,9%) in a word reading task and 42/45 (93,3%) in a non word reading task (BADA). He showed difficulties in the recognition of letters in the Incomplete letters task (VOSP) and in the *figure*-ground *segmentation* test. In addition he showed constructional and a very mild ideomotor apraxia and memory deficits. The performance on phonemic and semantic controlled associations remained within the normal range.

Table 1
Results of 99mTc –ECD SPECT in the two patients. Voxel-based analysis with SPM99 (Wellcome Department of Cognitive Neurology, Londra, UK) implemented in MATLAB 6.1 (Mathworks, Sherbon, MA). Comparisons between each patient and a control group (19 subjects) were made using $t$-statistics with appropriate linear contrasts. The tables report clusters above a statistical threshold set at $p < 0.05$ FWE corrected in MNI (Montreal Neurological Institute) space. **A**: patient L.P.; **B**: patient R.R.; **H**: hemisphere

**A**

| H | Regions | cluster extent | T | x | y | z |
|---|---|---|---|---|---|---|
| L | Inf/Mid Temporal Gyrus | 1825 | 8,74 | −46 | −58 | −4 |
| L | Middle Temporal Gyrus | | 8,62 | −48 | −50 | 0 |
| L | Middle Occipital Gyrus | | 7,11 | −40 | −70 | 0 |

**B**

| H | Regions | cluster extent | T | x | y | z |
|---|---|---|---|---|---|---|
| R | Middle Occipital Gyrus | 3908 | 10,46 | 32 | −78 | 16 |
| R | Middle Occipital Gyrus | | 10,38 | 36 | −70 | 20 |
| R | Angular Gyrus | | 9,64 | 40 | −60 | 32 |

### 2.2. *Case 2: R.R*

#### 2.2.1. *First examination*

A 61 year old right-handed woman with 13 years of education developed progressive visuo-spatial, concentration and memory difficulties, dressing apraxia, reading difficulties (she could not follow the correct line of print) and environmental disorientation over a period of three years. She evidenced great difficulties in putting objects in a cupboard, in laying the table, in getting into the car. Her family history was positive (her mother died with a diagnosis of probable AD).

Her MMSE score was 22/30. The neuropsychological assessment showed reading difficulties, constructional apraxia and visuospatial deficits. In addition, she showed a memory retrieval difficulty and a decline in verbal and semantic controlled associations. However, her language was fluent with good comprehension and repetition. Her executive abilities and insight were normal.

MRI (6 months before the first examination) showed minimal brain atrophy. The rCBF SPECT voxel-based analysis showed a large hypoperfusion centered in the right middle occipital gyrus and the right angular gyrus (Table 1).

In conclusion, R.R. had prominent symptoms due to parietal dysfunction, with a prevalent involvement of the right hemisphere.

#### 2.2.2. *Second examination*

One year later reading and visuospatial abilities had severely worsened and her spontaneous speech showed more anomias. Her MMSE was 15/30.

#### 2.2.3. *Third examination*

1 year later and 5 years into illness, she could not pour liquids or cut meat. She was spatially disoriented and she could not find the bathroom in her own house. She showed left visuospatial neglect, and her husband reported that the patient ate food only on the right side of the plate. Her MMSE score was 13/30. Neuropsychological assessment revealed ideomotor apraxia, acalculia and finger agnosia, alexia, constructional apraxia and memory deficits. The patient had turned into a complete Balint's syndrome. She was impaired in reaching towards visual targets and when asked to touch or reach objects placed at different locations within arm's reach. Optic ataxia was also evident in a test in which the patient had to mark circles. She put the marks only near the circles, never within them. Simultagnosia was evident in picture recognition, resulting in the misidentification of objects due to the fact that the patient's attention was captured by just one part of an object. For example, when she saw an elephant, she recognised only a pencil, pointing to the elephant's leg, or for the goose (in a lateral view), indicating the black eye, she identified a coffee bean. In addition R.R. was unable to follow rapid finger movements or to move the gaze voluntarily to different locations.

The two patients' data are summarised in Table 2.

## 3. Experimental study

### 3.1. *Control group*

Four normal subjects, matched to L.P. and R.R. for age and education (ages – 62 to 68-; education from 12

Table 2
Summary of clinical features at different examinations for L.P. and R.R.

| | L.P. Examinations | | R.R. Examinations | | |
|---|---|---|---|---|---|
| | I (8 months) | II (4 years) | I (3 years) | II (4 years) | III (5 years) |
| Enviromental Disorientation | − | − | + | + | + |
| Balint's Syndrome | − | − | − | nt | complete |
| Visuospatial Neglect | − | − | +/− | +/− | + |
| Gerstmann's Sindrome | acalculia | acalculia | acalculia | acalculia | acalculia/finger agnosia |
| Dressing Apraxia | − | − | + | + | + |
| Contructional Apraxia | − | + | + | + | + |
| Ideomotor Apraxia | − | +/− | − | + | + |
| Alexia | + | + | + | + | + |
| Visual Agnosia | − | − | − | − | + |
| Prosopagnosia | − | − | − | − | − |
| Anomia | − | − | nt | nt | + |
| Figure/ground segmentation | − | + | nt | nt | nt |

+ = present, − = absent, +/− = sub-clinical, nt = not tested. I,II and III are respectively the first, second and third examination, in parenthesis is shown the duration of disease.

to 16 years), were tested as a control group for the first examination.

### 3.2. Materials and procedure

A total of 90 Italian words were selected, 14,4% with irregular stress. The words ranged in length between four and nine letters (10 words of 4 letters, 14 of 5 , 12 of 6, 11 of 7, 24 of 8 ad 19 of 9). Words with different number of letters were balanced for age of acquisition ($p = 0{,}249$), familiarity ($p = 0{,}271$), bigram frequency ($p = 0{,}505$) [6]. Stimuli were delivered using Presentation software (version 10.3, Neurobehavioral Systems Inc., Albany, CA, http://www.neurobs.com) via a laptop computer. Word stimuli were presented one at time in the centre of a computer monitor and were printed in uppercase Arial 42 point font. The stimuli were written in white on a black background. Each word was presented for 8000 msec. After such period the letter string disappeared and was followed by an inter-trial interval of 2850 msec, then another word was displayed. The stimuli were presented to the participants in a random order. The task was to read the target aloud as fast and accurately as possible. Reading responses were collected by a microphone connected to the laptop. All subjects underwent a training session to familiarize with the task.

## 4. Results

### 4.1. Accuracy

Analysis of responses revealed that only PCA subjects could not read words in 8 sec (miss) and made errors, while the controls made no errors.

#### 4.1.1. Case 1: L.P.

*First evaluation*

L.P. used an oral letter-by-letter (LBL) strategy only in the case of longer words. He made a few visual errors, which were visually similar to the test words and differed only by few letters (i.e. "ombrello" for "ombelico"), and one regularization error (i.e. "lampáda" for "lámpada").

*Second evaluation*

The LBL strategy was more evident with respect to the first period, however he made only a few errors.

#### 4.1.2. Case 2: R.R.

*First evaluation*

R.R. made neglect errors (i.e. "tonno" for "sonno" ) and one visual error.

*Second evaluation*

She made 11 errors and 21 misses. The types of errors were more heterogeneous with respect to the first assessment. The patient showed derivational (i.e. "bombardamento" for "bomba"), visual (i.e. "maglia" for "medaglia"), neglect errors and perseverations. Furthermore she made 3 conduites d'approches. LBL reading was observed for few words.

*Third evaluation*

At this point reading was severely impaired. She made 33 errors and 46 misses. She made 19 neglect errors (i.e. "finto" for "soffitto", "lazzo" for "palazzo"). For 12 items she could identify only a few single letters and not the whole word, in particular for 9 items she could not identify letters on the left side of the word.

Table 3
Rates of different types of errors at different examinations for L.P. and R.R.

| Type of error | L.P. I (8 months) | L.P. II (4 years) | R.R. I (3 years) | R.R. II (4 years) | R.R. III (5 years) |
|---|---|---|---|---|---|
| Neglect | | | 4,44% | 4,44% | 21,11% |
| Visual | 3,33% | 3,33% | 1,11% | 2,22% | 1,11% |
| Derivational | | | | 3,33% | |
| Perseveration | | | | 2,22% | |
| Regularization | 1,11% | | | | |
| Miss | | 2,22% | 2,22% | 23,33% | 51,11% |
| Single letters identification (neglect) | | | | | 10% |
| Single letters identification | | | | | 3,33% |
| Tot % errors | 4,44% | 5,55% | 7,77% | 35,55% | 86,66% |

I,II and III are respectively the first, second and third examination, in parenthesis is shown the duration of disease.

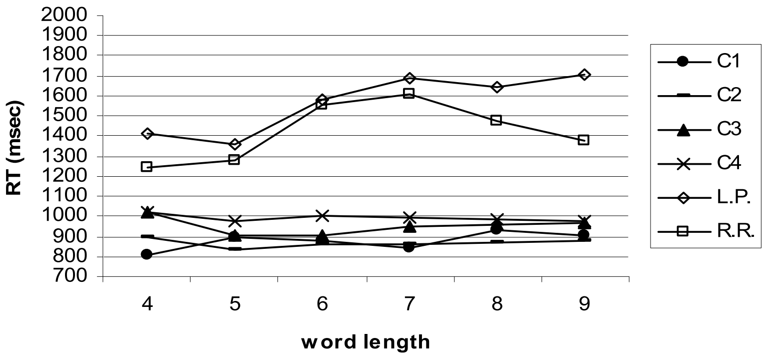

Fig. 1. Response times (msec) of L.P., R.R. and their matched controls as a function of word length. Note that the figure reports performances measured at the first examination for both patients corresponding to 8 months from the disease onset for patient L.P. and three years for patient R.R..

For example for "castagna" she said "s t r" , and for "stalla", only "a". In 2 of the 12 single letters identification she produced an automatic sequence ("a b c"). In addition she made only a visual error. Table 3 shows the percentage of different types of errors for L.P. and R.R..

### 4.2. Reaction times

Reaction times (RTs) more than 3 standard deviations away from the subject's average in a given condition were considered outliers, corresponding to 0,56% of the patients' data. We excluded from the analysis RTs corresponding to errors, conduites d'approches and distractions (15,28%). We did not analyze RTs for R.R. on the third evaluation as she gave only 11 correct responses. Rts means and standard deviations for both periods and for each condition are reported in Table 4.

#### 4.2.1. First examination

L.P. ($t$ = 3,937, one tailed $p$ = 0,05) [10] and R.R. ($t$ = 3,017, one tailed $p$ = 0,05) [10] were slower than controls, and L.P. more than R.R. ($t$ = 2,406; $p <$ 0,05). Controls participants and R.R. did not show a word length effect, however L.P. showed a very mild linear trend, with slower responses for more number of letters. A linear regression analysis of Rts as a function of word length indicated that each additional letter increased response time in L.P. by 68,6 msec (F(1,84) = 6,494; $p < 0.05$) (see Fig. 1).

#### 4.2.2. Second examination

No difference was detected between the two patients in terms of RTs ($t$ = 0,482, $p$ = 0,632). A paired sample T test showed longer Rts for the second examination than for the first for L.P. (t(78) = −10,156; $p <$ 0,001) and for R.R. (t(47) = −6,778; $p <$ 0,001). A linear regression analysis of Rts as a function of word length showed that each additional letter increased response time in L.P. by 300 msec (299,9 msec (F(1,81) = 27,331; $p < 0.0001$) see Fig. 2). R.R instead showed no significant effect of word length.

## 5. Discussion

We described two patients with a clinical diagnosis of PCA, characterized by different clinical features, focusing on the evolution of their reading disorders.

Table 4
Reaction times (RTs) mean for L.P. and R.R. for both periods and for each condition

| | RTs mean (sd) | | | |
|---|---|---|---|---|
| | L.P. I (8 months) | L.P. II (4 years) | R.R. I (3 years) | R.R. II (4 years) |
| 4 letters | 1408 (446) | 1688 (540) | 1243 (217) | 5051 (685) |
| 5 letters | 1361(241) | 2121 (536) | 1282 (327) | 1984 (547) |
| 6 letters | 1581(496) | 2834 (1206) | 1556 (395) | 3308 (1824) |
| 7 letters | 1690 (623) | 2927 (288) | 1608 (820) | 2148 (1025) |
| 8 letters | 1647(348) | 2956 (996) | 1474 (402) | 2775 (997) |
| 9 letters | 1703 (465) | 3341 (1096) | 1374 (157) | 2587 (1153) |
| Tot | 1580 (440) | 2724 (1029) | 1427 (478) | 2686 (1262) |

I,II and III are respectively the first, second and third examination, in parenthesis is shown the duration of disease.

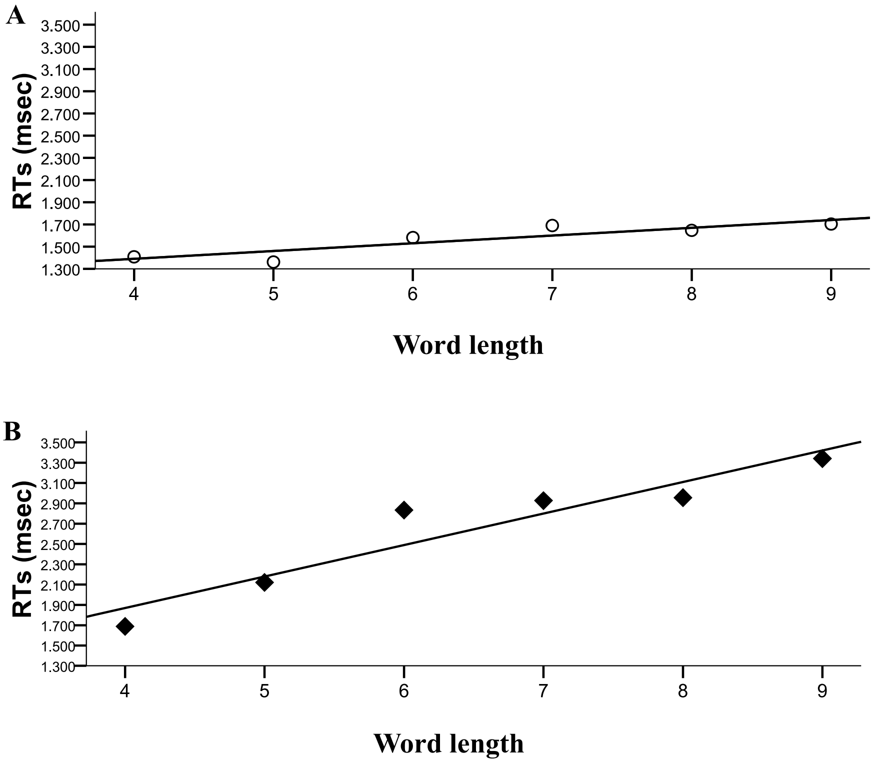

Fig. 2. Scattergrams showing the relationship between L.P. reaction times of words and word length for both periods. A = first examination corresponding to 8 months from the disease onset ; B = second examination corresponding to 4 years from the disease onset.

L.P. was followed from the early stages of disease. At an early stage he showed symptoms prominently due to the involvement of the left hemisphere, including deficits suggesting a dysfunction of ventral pathways (alexia) and inferior parietal lobe (acalculia). Additional symptoms appearing with disease progression reflect the further extension of the underlying pathology.

Specifically, starting from the first period of examination he showed a qualitative LBL reading and a mild word length effect. This pattern evolved during the course of the disease showing a strong effect of the 'number of letters' in the second period, with an increasing of 300 msec for additional letter. We may conclude that L.P. showed a 'letter by letter' alexia, which has been already reported in patients with Posterior Cortical Atrophy [1,9,13,14,16], and is typically associated with damage involving the temporo-occipital regions.

R.R., who was seen three years after symptom onset, initially showed symptoms which can be ascribed to right parietal dysfunction. However, at the time of her last examination she showed symptoms suggesting a severe involvement of parietal areas in both hemispheres (complete Balint's syndrome), as well as an involvement of the ventral areas (agnosia). At the first evaluation R.R. showed mild neglect alexia, while left hemineglect was not detected either in everyday life or at neuropsychological assessment. At an intermediate period the reading difficulties became more severe, showing slower latencies and difficulties in the identification of the words. At the time of this evaluation she showed different types of errors (visual, neglect, derivational), as well as many misses. In the last assessment neglect alexia as well as hemineglect were evident. Arduino et al. [4] reported that stimulus duration influences the types of errors in patients with neglect alexia. Timed and untimed presentation of the stimuli produced, respectively, more visual and more neglect errors. They suggested that "time-limited stimulus exposure may give leeway for early visual processing components, relatively unaffected by the rightward bias that characterizes unilateral neglect". In this study we used the same stimuli duration in three different periods in a PCA patient with an initial mild neglect alexia. The progression of the illness leads to different outcomes in terms of accuracy in the same patient. For example, in the second period R.R. showed both visual and neglect errors, while in the third period neglect errors became prominent (she made only 1 visual error). We can thus hypothesize that in the late stage of the disease the rightward bias could affect more severely the early visual processing components, capturing the patient's visual attention on the final rightward letters of the string.

In conclusion, PCA patients in the early stages of disease can be classified according to the prominent functional and neuropsychological involvement of the ventral vs. dorsal areas, and/or of the left vs. right hemisphere. Although in the late stage of the disease these different variants tend to merge, some qualitative aspects of alexia remain different. In the case of L.P., the evolution of the reading disorders is in line with a progressive involvement of the temporo-occipital cortex. In the case of R.R., who showed an initial, isolated neglect alexia, the progression of disease was possibly associated with extending, bilateral parietal and temporo-parietal dysfunction.

We can state that the qualitative pattern of reading impairment continues to remain a distinctive aspect of PCA also in advanced stages of the disease reflecting the initial underlying anatomical grounding.
